# From Kitchen to Cell: A Critical Review of Microplastic Release from Consumer Products and Its Health Implications

**DOI:** 10.3390/toxics14010094

**Published:** 2026-01-20

**Authors:** Zia Ur Rehman, Jing Song, Paolo Pastorino, Chunhui Wang, Syed Shabi Ul Hassan Kazmi, Chenzhe Fan, Zulqarnain Haider Khan, Muhammad Azeem, Khadija Shahid, Dong-Xing Guan, Gang Li

**Affiliations:** 1 State Key Laboratory of Regional and Urban Ecology, Ningbo Observation and Research Station, Institute of Urban Environment, Chinese Academy of Sciences, Xiamen 361021, China; mr.ziaa69@gmail.com (Z.U.R.); chwang@iue.ac.cn (C.W.); hkazmi@iue.ac.cn (S.S.U.H.K.); czfan@iue.ac.cn (C.F.); zulqarnainhaiderkhan@gmail.com (Z.H.K.); azeem@iue.ac.cn (M.A.); gli@iue.ac.cn (G.L.); 2 Zhejiang Key Laboratory of Pollution Control for Port-Petrochemical Industry, CAS Haixi Industrial Technology Innovation Center in Beilun, Ningbo 315830, China; 3 University of Chinese Academy of Sciences, Beijing 100049, China; 4 Istituto Zooprofilattico Sperimentale del Piemonte, Liguria e Valle d’Aosta, 10154 Torino, Italy; paolo.pastorino@izsplv.it; 5 Center for Agricultural Resources Research, Institute of Genetics and Developmental Biology, Chinese Academy of Sciences, Shijiazhuang 050021, China; khadijaashahid69@mails.ucas.ac.cn; 6 State Key Laboratory of Soil Pollution Control and Safety, Zhejiang Provincial Key Laboratory of Agricultural Resources and Environment, College of Environmental and Resource Sciences, Zhejiang University, Hangzhou 310058, China; dxguan@zju.edu.cn

**Keywords:** plastic contamination, toxicity, trophic transfer, consumer products, direct leaching, food safety

## Abstract

Microplastics (MPs) are pervasive environmental pollutants, widely distributed from aquatic ecosystems to the terrestrial food chain, and represent a potential route of human exposure. Although several reviews have addressed MP contamination, a critical synthesis focusing on pathways through which consumer goods directly enter food and beverages, along with corresponding industry and regulatory responses, is lacking. This review fills this gap by proposing the direct release of MPs from common sources such as food packaging, kitchen utensils, and household appliances, linking the release mechanisms to human health risks. The release mechanisms of MPs under thermal stress, mechanical abrasion, chemical leaching, and environmental factors, as well as a risk-driven framework for MP release, are summarized. Human exposure through ingestion is the predominant route, while inhalation and dermal contact are additional pathways. In vitro and animal studies have associated MP exposure to inflammatory responses and oxidative stress, neurotoxicity, and genomic instability as endpoints, though direct causal evidence in humans remains lacking, and extrapolation from model systems necessitates caution. This review revealed that dietary intake from kitchen sources is the primary pathway for MP exposure, higher than the inhalation pathway. Most importantly, this review critically sheds light on the initiatives that should be taken by industries with respect to global strategies and new policies to alleviate these challenges. However, while there has been an upsurge in research commenced in this area, there are still research gaps that need to be addressed to explore food matrices such as dairy products, meat, and wine in the context of the supply chain. In conclusion, we pointed out the challenges that limit this research with the aim of improving standardization; research approaches and a risk assessment framework to protect health; and the key differences between MP and nanoplastic (NP) detection, toxicity, and regulatory strategies, underscoring the need for size-resolved risk assessments.

## 1. Introduction

**Figure 1:**
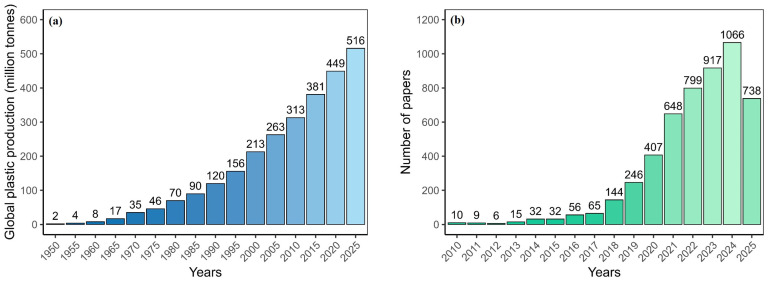
(**a**) Global plastic production, 1950 to 2025, reflecting the potential rise in plastic manufacturing that increases MP pollution; (**b**) annual number of scientific research papers on MPs in food and human exposure from 2010 to present, documenting the fast progress of research in this field.

Plastics have become indispensable in modern society due to their cost-effectiveness, chemical resistance, and mechanical durability [1]. The production of plastics has been increasing gradually every year globally and is certainly accompanied by rapid industrialization and urbanization [2], triggering the production of 415 million tons every year by 2023 [3], marking a 63% increase over 2010 production (Figure 1a). Notably, only 21% of plastics are either recycled and/or incinerated worldwide, with the remaining 79% persisting in landfills [4,5]. Incorrect dumping and mishandling of plastic waste produces tiny particles, <5 mm in diameter, called microplastics (MPs) [6], which undergo further physical, biological, and photodegradation to form primary and secondary MPs [7,8,9]. It is crucial to differentiate MPs (typically 1 µm to 5 mm) and nanoplastics (NPs, <1 µm) [6,10], as they reveal significant differences in terms of detection methods, environmental behavior, toxicokinetics, and potential health impacts. Although this review focuses primarily on MPs due to the more extensive availability of relevant exposure data, NPs, which are often released concurrently with MPs, pose unique challenges in terms of detection and risk assessment.

MPs function as representatives of prevalent pollutants in the environment that pose imminent threats to the ecosystem and human health [11,12]. Their micro-scale features, such as dimensions that favor surface area to volume and hydrophobicity, among others, facilitate adsorption and transport of accompanying contaminants, such as persistent organic pollutants/heavy metals/pathogenic microorganisms [13,14]. MPs have infiltrated various environmental matrices, food and freshwater systems, and even human stools [15,16]. Subsequently, the focus in scientific research has steadily shifted to direct human exposure pathways, such as MP ingestion from food and drinks [17,18].

Of these pathways, the direct release of MPs from food and beverages and kitchenware establishes an instant and major route for human exposure. Permeating items like bottled water, plastic tea bags, breast milk storage bags, infant feeding bottles, and cooking utensils are now identified as MP release sources under thermal stress, mechanical agitation, and prolonged use [19,20,21]. This “direct-from-product-to-food” route accelerates long-term human exposure, which is postulated to be one of the factors affecting adverse health impacts that are noticed in models such as gastrointestinal inflammation, endocrine dysfunction, and oxidative stress [22]. The vulnerable population of infants and children is also negatively impacted due to prolonged handling with plastic baby bottles during development stages [21].

Although the existence of the MPs is well established within current studies [15,23,24], there exists a need for an extensive and critical review covering the direct leakage of consumer products and kitchen wares. Furthermore, the reaction to this issue within industries and governments has often taken the wrong track. This review seeks to mend this gap by presenting an innovative consolidation of signs within the unbroken contamination cycle of MPs. It offers added value with its unique emphasis on highly relevant references and a detailed investigation of combined release mechanisms and the current environment of mitigation measures. Therefore, the fundamental aims and objectives of this review were to (a) explore the mechanism and magnitude of MP release from the most prevalent food contact substances and usages in the kitchens, (b) critically evaluate the potential impact caused by the direct contamination route on human health, and (c) substantially assess the reactions within industries to counteract MP pollution caused by these substances. Furthermore, a risk-driven conceptual framework for MP release based on material fragility and stress intensity has been proposed in this review.

## 2. Research Trend and Scientometric Analysis

A systematic search was conducted on the Web of Science Core Collection (WOSCC) database to comprehensively identify relevant studies using keywords such as (“microplastic” OR “microplastics” OR “plastic particle”) AND (“food” OR “beverage” OR “drink” OR “kitchenware”) AND (“human health” OR “health effect” OR “ingestion” OR “consumption” OR “contamination” OR “pollution” OR “exposure” OR “detection”) to quantitatively map the logical structure of investigation on MPs in human food chain. The analysis documented a noticeable increase in publications from 2014 to 2024 (Figure 1b), emphasizing the speedy rise of this field at the intersection of environmental science and public health.

**Figure 2:**
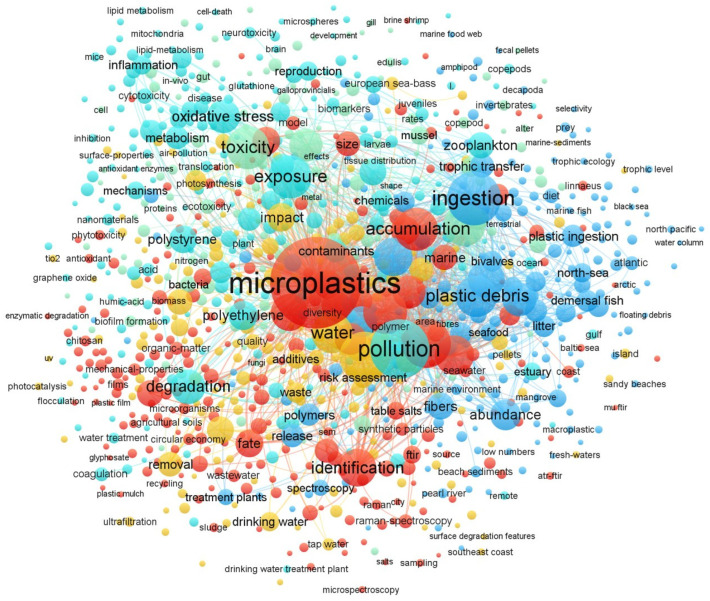
Network map of co-occurrence keywords on MPs related to food and human exposure. Node size demonstrates the regularity of keywords; meanwhile, line width reflects the strong points of co-occurrence among the terms.

An illustrative network map of co-occurrence keywords was generated using the VOSviewer software (version 1.6.20) to visualize the intangible interactions within these research fields. This keyword co-occurrence network map (Figure 2) not only shows research hotspots but also reveals the structure of the current knowledge system. We can clearly see several closely connected research clusters. One cluster, focused on “food chain”, “packaging”, and “detection”, centers around food science and analytical chemistry. Another cluster, centered on “toxic effects”, “cell damage”, and “health impacts”, is related to toxicology and medicine.

However, what is crucial is that the integrative research linking these two core clusters, acting as a “bridge”, especially reviews tracking the full chain of MPs from “kitchenware wear” (physical–chemical processes) to “cellular stress” (biological consequences), shows weaker connections in the network. Existing reviews either focus on environmental sources or specific health outcomes, but none fully integrate “release mechanisms”, “multi-pathway exposure assessments”, and “cross-scale health effects”.

## 3. Sources and Pathways of MPs in Food Containers

**Figure 3:**
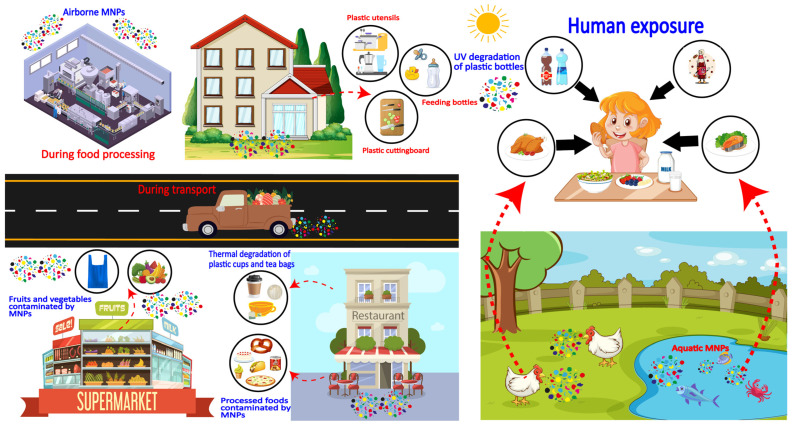
Possible MP sources and pathways into human food. The figure exemplifies the causative factors that promote the migration of MPs into the food chain. Fundamental sources comprise food processing, packaging material, thermal degradation, chemical leaching, and mechanical abrasion.

The release of MPs from materials in contact with food is not a homogeneous process but is instead regulated by specific physical and chemical mechanisms. Figure 3 illustrates the primary source and multidimensional mechanism leading to the release of MPs into food and drinks. Understanding these dominant mechanisms is crucial for predicting exposure risks and developing targeted mitigation strategies. This section systematically categorizes and reviews common sources of MP pollution based on three dominant release mechanisms: thermal stress, mechanical abrasion, and chemical/environmental factors.

### 3.1. MP Release Sources Driven by Thermal Stress

High temperatures can significantly accelerate polymer degradation, resulting in the detachment of MPs [25]. This mechanism is mainly important for products that are frequently exposed to heat during their usage. For instance, polypropylene (PP) feeding bottles release up to 16.2 million MPs/L during formula preparation when subjected to high-temperature sterilization (70–100 °C) and vigorous shaking, translating to an estimated daily intake of 1.58 million PP particles for 12-month-old infants [21].

Plastic tea bags release exceptionally high MPs during routine brewing; a single tea bag releases up to 11.6 billion MPs + 3.1 billion NPs at 95 °C [26]. However, these findings have been challenged due to methodological limitations that may lead to overestimation, as not all observed particles were chemically confirmed as plastic [27]. These extreme values demonstrate the results of a single laboratory study conducted under standardized conditions and may not reflect the average exposure levels in real-world scenarios, which depend on factors like brand, brewing habits, and analytical recovery rates. Therefore, it is essential to fully acknowledge the high variability among different studies when interpreting exposure estimation results. Regarding commercial brands (518,459 ± 136,440 MPs/teabag [28], Iran), an annual MP release ≥10.9 million grams was recorded from tea bags, and these release mechanisms are most probably caused by thermal decomposition at high temperatures (≥70 °C), physical peeling during immersion, and water absorption weakening polymer integrity [28,29]. However, 94% of commercial filter bags (poly-ethylene terephthalate (PET), PP, and nylon) shed MPs (620–840 µm fibers) within 5–30 min of brewing [29]. This pathway shows how routine food preparation transforms packaging into acute MP exposure sources.

Takeout food containers and other packaging used to hold hot food are also significant sources of MPs. The rapid expansion of the takeout food service industry has led to a surge in the consumption of these containers, which are typically made of poly-styrene (PS) or PP [30]. These containers degrade into MPs when they come into contact with hot food. Studies have found considerable MP release from frequently used packaging materials, and individuals who eat takeaway food regularly may consume from 12 to 203 particles of MPs/week through their takeaway containers alone [31].

Similarly, paper cups with plastic additives show temperature-dependent emission peaks when exposed to near-boiling temperatures [32], highlighting that heat can potentially promote MP release from composite materials. For instance, plastic-lined containers can release a small number of particles at room temperature but spike into the thousands of MPs per serving when filled with hot beverages (85–90 °C) [33,34]. There are also significant differences between manufacturers and product brands, mainly due to variations in the quality of plastic lining (such as thickness and polymer type) and manufacturing standards [35]. The products that could be considered the most “dangerous” are those that expose consumers to high thermal stress with a fragile or thin polymer matrix, such as plastic tea bags or single-use containers for hot food, which can release hundreds of millions of particles per use [36]. To limit exposure, consumers can stay away from hot liquid storage in plastic-lined containers or use alternatives like ceramic cups and/or stainless-steel cups and let the beverages cool before shifting them into single-use containers [37].

### 3.2. MP Release Sources Driven by Mechanical Abrasion

The primary dynamic force of MP release from kitchenware and packaging material involves physical forces and friction in use. Here, routine food preparation activities result in the physical degradation of the polymer surface directly.

For instance, plastic cutting boards can release significant numbers of MPs during food preparation due to mechanical abrasion. Yadav et al. (2023) [38] estimated MP emission from PP and polyethylene (PE) cutting boards during ingredient processing, revealing that PP boards shed 14–71% more particles and 5–60% greater mass than PE boards under identical cutting conditions, with harder vegetables like carrots generating higher amounts (Table 1). This release takes place primarily through the direct physical interaction between knives and board surfaces, where amplified force requirements on solid ingredients increase polymer fragmentation. Studies of the food market have validated this type of direct physical interaction: MP pollution in chicken (0.03 ± 0.04 to 1.19 ± 0.72 particles/g) and fish (0.014 ± 0.024 to 2.6 ± 2.8 particles/g) was directly associated with the degree of wear on cutting boards [39]. Plastic-based kitchen appliances, such as blenders, rank among significant sources of MP contamination based on material composition and operational mechanism. These appliances are equipped with high-torque motors that power stainless-steel blades within polymer containers, thus efficiently processing complex substances, including ice, frozen ingredients, and coffee beans.

High-torque kitchen appliances have been considered a significant source of MP contamination due to their operational mechanisms [40]. Recent experimental work has revealed that conventional blending operations release considerable quantities of MPs and NPs: Luo et al. (2023) [41] recorded 0.36–0.78 × 10^9^ MP and NP particles emissions within only 30 s at ambient temperature. This happens mostly through two different mechanisms occurring simultaneously, which are direct abrasion between the rotating blades and container walls and the mechanical fatigue of plastic components subjected to high-shear hydrodynamic forces, as stated by Snekkevik et al. (2024) [42]. Intense rotational velocities ranging from 15,000 to 30,000 RPM induce greater stress on the polymer structure, causing material fragmentation that increases with ingredient hardness and processing time [43]. Therefore, it is not estimated but clearly evidenced from these findings that blenders are powerful emitters of MPs.

Beyond single-use abrasion, repeated-use-triggered damage also contributes to the significant release of MPs. Likewise, various sterilization methods, reusing frequencies, and mechanical activities (e.g., bottle opening and closing and cap twisting) extensively cause the release of MPs [18,44,45]. Similarly, repeated use has shown that feeding bottles and water bottles subsequently release 53 ± 9.4 to 393 ± 57.5 MPs/mL and 100 to 209 MPs/mL, respectively; however, this depends on the usage, resulting in irregular/spherical particles with diameters of 20–500 µm. Secondary exposures through plastic syringes also release 1.74 MPs in every single injection [20]. Conversely, safe usage methods could partially minimize these risks. For example, with reusable bottles and containers, once used, high-pressure washing should be able to remove loose debris. Furthermore, the use of more durable materials, such as glass or stainless steel, may control polymer degradation [46].

### 3.3. MP Release Sources Driven by Chemical and Environmental Factors

The slow degradation process, which is influenced by chemical interactions and environmental conditions, can also lead to the release of MPs over time, particularly in products that have not been subjected to extreme stress. The process of bottling water and long-term storage has shown that chemical leaching and continued material aging can initiate the release of MPs. Global studies confirm MPs in bottled water across diverse brands and regions (Table 1). Zuccarello et al. (2019) [47] detected MPs in 100% of 30 commercial mineral water samples (avg.: 5.42 pcs/L; mean: 2.44 µm). Similarly, in China, 2–23 MPs/bottle were found across 23 brands [48]; in Malaysia, an average of 11.7 MPs/L was found in 8 brands, primarily from packaging/caps [18]; and 93% of 259 samples were contaminated in 9 different brands from different countries [49]. This ~93% plastic-bottled water contamination was significantly higher than that of the public drinking water source (tap water) [50]. Therefore, human exposure is considerable, and in other beverages (soft drinks and beers), it is up to 28 MPs/L [51]. One study found that daily intake via bottled water in adults is ∼1.53 million MPs/kg bodyweight/day, and in children, it is ∼3.35 million MPs/kg bodyweight/day [47], but this study has been critically questioned due to methodological limitations in particle identification and quantification [52], which may inflate reported exposure levels relative to other studies. Similarly, packaged ice is an important carrier of MPs, and its source of pollution is the environment and processes. MPs detected in commercial ice (e.g., 19 ± 4 to 178 ± 78 items/L) are mainly from drinking water sources used for production, suggesting that MP contamination from the environment can enter beverages directly through this pathway [53]. The freezing process itself does not produce MPs but instead concentrates the particles already present in the water. This highlights the fact that MP exposure can occur in products that are already contaminated during production, regardless of how consumers use them. Long-term contact between water and plastic packaging can lead to progressive leaching. Thus, bottled water can be predicted as a major pathway of direct human MP intake. The duration of storage can further aggravate degradation; storage in refrigeration or at room temperature for over six months can gradually degrade plastic material and increase the release of MPs [43]. Secondary MP formation is also possible through plastic sealing film and bottle caps in non-plastic containers, glass bottles, and paper boxes [19,54]. Chemical degradation of plastics results in MP release due to hydrolytic, oxidative, and ion-mediated pathways. Aqueous chemistry plays an important role in modulating these processes; common ions (Ca^2+^, HCO_3_^−^, Fe^3+^, Cu^2+^) and particulate matter (e.g., Fe_2_O_3_) can form passive surface complexes that hinder MP release over time by building up protective mineral coatings on polymer surfaces [55]. Moreover, slow chemical leaching occurs during storage, and due to the aging of materials, the treatment processes for wastewater and sewage may also indirectly stimulate the formation of secondary MPs/NPs through the partial oxidation and degradation of polymers [56,57]. Advanced oxidation processes (AOPs), including Fenton and photo-Fenton reactions, ozonation, and ultraviolet-based treatment technologies, produce reactive free radicals that oxidize polymer surfaces, weaken carbon–carbon bonds, and initiate chain breaks, leading to polymer aging [58]. This oxidative weathering process accelerates the instability of polymers, making them more prone to fragmentation into smaller particles. Subsequently, treated water that is reused for irrigation or as a source of drinking water may become a significant indirect pathway for chemically aged MPs into food and beverages [59,60].

On the other hand, water pH has a dual role in contamination, where a low pH accelerates polymer hydrolysis caused by proton-catalyzed chain scission, and alkaline conditions simply cause oxidative degradation. MP release rates inversely relate to plastic density and directly relate to material thickness, as thinner, low-quality polymers demonstrate higher potentials for emissions [61]. As an example of this, micro-structural breakdowns exhibit large material differences, as tested in different forms of honey packaging, where amorphous regions facilitated quicker MP release compared to crystalline domains [62].

**Table 1:** Occurrence of MPs in beverages, food items, and kitchen/home utensils with respect to primary release mechanism and exposure frequency.

Dietary Source	Primary Release Mechanism	Typical Polymer Types	Estimated MP Particles Per Serving	Identification and Quantification	Exposure Frequency	Methodological Limitations	Confidence Level	References
Bottled water	Chemical leaching/mechanical abrasion	PE, PP, PS, PVC, PU, PET, PA, PEVA, PAA, cellulose	2 to 23 particles/bottle	μ-FTIR	EDI 0.274 MPs/kg/d	Limited sample size, inter-brand variability not fully captured	Moderate	[48]
Soft drinks	Packaging leaching	PEA, ABS, PA	40 ± 24.53 particles/L	μ-Raman spectroscopy	-	Raman bias toward larger particles	Moderate	[51]
Drinking water	Chemical leaching/environmental factors	-	656.8 ± 632.9 μg of MPs/L	SEM-EDX	EDI 1.53 million MPs/kg bodyweight/day in adults, 3.35 million MPs/kg bodyweight/day in children	Mass-to-particle conversion uncertainty, SEM-EDX lacks polymer confirmation	Low	[47]
Bottled water	Mechanical abrasion and bottle caps twisting	PET, PP	11.7 ± 4.6 particles/L	Membrane filtration method	EDI 0.089 to 0.25 MPs/kg/day in children, 0.068 to 0.19 MPs/kg/day in adults	Limited particle size range, bottle handling conditions may vary	Moderate	[18]
Energy drinks	Packaging leaching	PA, PEA	14 ± 5.79 particles/L	μ-Raman spectroscopy	-	Small sample size, limited polymer confirmation	Moderate	[51]
Laurentian Great Lakes Beer	Product processing	-	4.05 particles/L	FTIR	EDI 5800 particles/year	Low concentrations near detection limits, limited brands analyzed	Moderate to high	[50]
Tap water	Environmental factors	PS, PVC, PA	1.67 to 2.08 μg of particles/L	FTIR	-	Conversion from mass to particle uncertain	Moderate	[63]
Beer	Packaging leaching	PET, PEA, PA	152 ± 50.97 particles/L	μ-Raman spectroscopy	-	Raman size bias, limited geographic scope	Moderate	[51]
Ice cubes	Mechanical abrasion/food processing	PP, PE, PVC, PVA, cellophane	19 ± 4 to 178 ± 78 MPs/L	SEM-EDX	EDI 490 to 10,000 MPs/year	Freezing and handling may exaggerate release	Low to moderate	[53]
Cold tea	Packaging leaching	PEA, PA	11 ± 5.26 particles/L	μ-Raman spectroscopy	-	Limited brands and preparation styles	Moderate	[51]
Tap water	Environmental factors	-	61 particles/L	FTIR	-	Regional sampling only	Moderate to high	[50]
Infant formula	Packaging	PA, PE	42 ± 27 MPs/100 g	μ-Raman spectroscopy	EDI 49 ± 32 MPs/day	Heating conditions may exceed household practice, sensitive population	High	[64]
Beef hamburgers	Food processing/packaging	PP, PE, PC	200 to 30, 300 MPs/kg	μ-FTIR	-	Broad range reflects heterogeneous processing conditions	Moderate	[65]
Fish	Food processing/packaging/environmental factors	PA, PET, PE, PS, PP, PVC, PMMA, ABS	11 ± 16 to 25 ± 50 MPs/fish	μ-FTIR	-	Species-dependent variability	Moderate to high	[66]
Commercial sea salt	Food processing/packaging/environmental factors	-	46.7 to 806 particles/kg	FTIR	-	-	-	[50]
Canned fish	Canning processing/cleaning process	PET, PS, PP, PS-PP, PS-PET, nylon, PVC, LDPE	25.60 ± 0.87 items/cans	μ-Raman spectroscopy, SEM-EDX	EDI 44 to 1126 MPs/month in adults, 20 to 512 MPs/month in children	Thermal and mechanical stress may amplify release	Moderate	[67]
Take-away food	Thermal stress	PP, PS	3 to 29 items/container	ATR-FTIR, SEM	EDI 12 to 203 items/ person/week	Heating conditions represent maximum plausible exposure	Moderate	[31]
Food containers	Thermal stress	PP, PE, PVC, PET, PA, PU, PS	29 to 552 items/container	μ-FTIR	EDI 145 to 5520 MPs/month	Repeated heating exaggerates release	Moderate	[19]
Infant feeding bottles	Thermal stress + mechanical abrasion	PC, PP, PPSU	53 ± 9.4 to 393 ± 57.5 particles/mL	μ-FTIR, LDIR	-	Controlled lab conditions may exceed daily use	High	[20]
Breastmilk storage bags	Thermal stress + chemical leaching	PE, PET, nylon-6	0.61 to 0.89 mg of MPs/day	μ-Raman spectroscopy	EDI 0.61 to 0.89 mg/day	Limited brands analyzed	Moderate	[68]
Plastic teabags	Thermal stress	Nylon, PET	16 μg of MPs/cup of tea	FTIR, XPS	-	Overestimated values, observed particles not confirmed as plastic	Low to moderate	[26]
Plastic bowls	Mechanical abrasion + thermal stress	PP, PS, PE, ABS, SAN, melamine	331 to 898 particles/bowl	FTIR, Py-GC/MS	-	Aggressive abrasion scenarios	Moderate	[69]
Cutting/ chopping boards	Mechanical abrasion	PE, PP	49.5 to 50.7 g of MPs/ person/year	FTIR	EDI 72 to 79.5 million MPs/person/ year	Assumes frequent, high-intensity use	Low to moderate	[38]
Kitchen blenders	Mechanical abrasion	ABS, PS	0.36 to 0.78 billion MPs/30 s blending	Raman spectroscopy, SEM	-	Short-term extreme stress test	Low	[41]
Plastic cutting boards	Mechanical abrasion	-	0.03 ± 0.04 to 1.19 ± 0.72 particles/g in chicken, 0.014 ± 0.024 to 2.6 ± 2.8 particles/g in fish	FTIR	-	Transfer to food matrix uncertain	Moderate	[39]
Teabags	Thermal stress	PET, nylon, cellulose acetate	518,459 ± 136,440 MPs/teabag	Raman spectroscopy, SEM	EDI 17,282 MPs/kg bodyweight/day in children, 14,813 MPs/kg bodyweight/day in adults	Upper-bound experimental scenario	Low to moderate	[28]
Non-stick cooking pan	Mechanical abrasion + surface degradation	-	9100 particles/30 s stirring	μ-Raman spectroscopy, SEM	-	Surface damage exaggerates release	Low	[70]
Dish sponges	Fragmentation + mechanical abrasion	PET, nylon PA6	100 to 200 items/sponge	PCA-Raman spectroscopy, SEM	-	Fragment transfer pathway uncertain	Moderate	[71]

Note: The sign “-” indicates unspecified. Recorded MP concentrations are highly method-dependent. Variability (e.g., standard deviation) is omitted where not provided in the original study, limiting comparability. Moreover, reported exposure values should not be interpreted as a uniform daily intake. Several studies intentionally applied extreme thermal/mechanical stress to simulate upper-bound release scenarios, while others reflect typical real-world consumption. Due to methodological limitations, we assigned high, medium, or low confidence levels to reflect data reliability rather than absolute exposure estimates.

### 3.4. Risk-Driven Framework for MP Release

**Figure 4:**
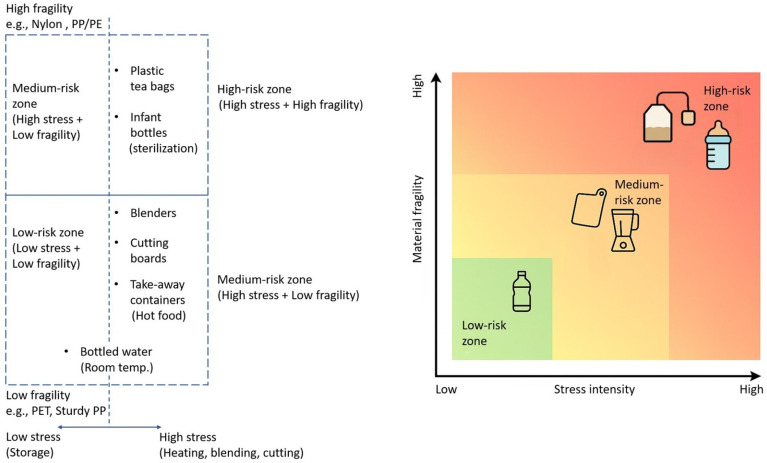
A conceptual risk-driven framework of MP release from food contact materials. This model considers the relative risk of exposure by plotting the stress intensity during use to the integral sensitivity of the polymer material. The position of each item is based on a synthesis of the data existing in this review: the closer to the top right corner of the diagram, the higher the potential risk of MP release.

The quantitative exposure estimation results presented in Table 1 and throughout this review are primarily due to differences in analytical methods (such as detection limits and the range of particle sizes considered), product brands, usage conditions, and study designs. Consequently, direct comparisons between different studies are challenging. Many of the estimation results lack robust uncertainty quantification, while some extreme values (such as the release of billions of particles per serving) stem from specific experimental protocols and may overestimate the release rates under typical usage. Future research urgently needs to standardize exposure modeling and reporting, including the provision of critical information, such as standard deviations, recovery rates, and detection limits, to enhance the reliability of risk assessments. The relative risk of MP release from common food contact materials can be integrated into a conceptual model (Figure 4) based on the interaction between the stress intensity and material properties. This two-dimensional matrix evaluates products by comparing the intensity of external stress encountered during typical use (such as thermal stress, mechanical abrasion, and chemical/environmental leaching) with the inherent fragility or degradation sensitivity of the polymer material. This framework reveals different risk areas: the closer a product is to the upper right corner of the chart, the greater the likelihood of releasing significant numbers of MPs. For example, plastic tea bags (high heat stress + fragile polymer matrix) and baby bottles during disinfection (extreme heat and mechanical stress) are of particular concern due to this synergistic effect. On the other hand, items like plastic cutting boards and blenders (typically made from PP and PE) are subject to high mechanical stress, thus falling into the medium-risk category. Products like bottled water stored at room temperature are in the low-risk zone, as the stable PET material experiences minimal stress. This model provides valuable heuristic guidance for prioritizing mitigation measures, indicating that the greatest potential for reducing human exposure lies in addressing high-risk applications where both harsh use conditions and material sensitivities are present.

Thermal stress steadily leads to the release of a significant quantity of particles. For example, practices such as the brewing of plastic tea bags or the high-temperature sterilization of baby bottles can result in the release of tens of billions to trillions of particles with each use [21,26]. These released particles usually involve a complex mixture of micro- and nano-sized fragments that are generated through the fast degradation of the polymer matrix [72]. This mechanism is mainly hazardous because the high-energy input simultaneously promotes the release of both polymer additives, such as plasticizers and stabilizers, and the monomers themselves [73], leading to a significant mixed effect that results in both physical and chemical exposures.

Compared to thermal stress, the MP particles generated by mechanical wear are normally low, but this still establishes a high-frequency and extensive exposure pathway. Common kitchen utensils, such as cutting boards and mixers, may release millions to billions of MPs under normal use conditions [38,41]. These particles have larger sizes and irregular shapes and are usually of a fibrous structure; thus, they are at great risk of causing physical abrasion to gastrointestinal tissues. Although the possibility of leaching additives is lower in this pathway compared to thermal degradation-generated particles, the amount of plastic that enters the diet through this pathway is significant, and constant physical stimulation can disrupt intestinal homeostasis and threaten health [74].

Chemical and environmental leaching results in a slow but long-lasting process, ubiquitous in nature due to the dependence on packaged and bottled foods around the world. Typically, weeks or months of storage release a robust but lower number of MP particles through chemical degradation [18,47]. These types of MPs have a regular form and a smaller size. Chronic and low-dose ingestion of MPs due to food material contact over longer periods is the main risk of this exposure pathway [75]. These particles increase the slow migration of additives into food before release, leading to chronic chemical exposure and possible risks to health [76]. Therefore, it is quite clear that the highest-risk products are concentrated in the right quadrant, where high-intensity stress (such as high temperatures) causes materials to be susceptible to damage. This may lead to the release of particles, along with high potential chemical hazards. This viewpoint is crucial in guiding targeted mitigation measures in material design, regulatory development, and consumer behavior.

### 3.5. Critical Appraisal of Analytical Methodologies and Reported Data

The MP concentrations reported in Table 1 and cited throughout the text are basically influenced by the analytical techniques employed. Studies using µ-FTIR, µ-Raman, Py-GC/MS, and visual microscopy exhibit differences in detection limits, statistically measurable particle size ranges, the reliability of polymer identification, and sensitivity to false-positive results [77]. For example, spectral techniques can identify polymer types but may miss particles smaller than approximately 10–20 µm; thermal degradation techniques provide mass data but lose information on particle quantity and morphology [78]. The lack of a unified standardized process for sample pretreatment, contamination control (which is essential in the analysis of low-quality MPs), and data reporting (such as whether blank samples, background correction, and recovery rates are included) makes comparisons between different studies and conducting meta-analyses more complicated. The conclusions regarding high exposure levels attributed to certain specific products often rely on a single methodological approach, with a lack of independent verification using complementary analytical techniques. This methodological heterogeneity must be fully considered when assessing the strength of evidence for any particular exposure source. The particle size distribution is rarely reported in full detail, but it is crucial for understanding its relevance to toxicology. Smaller particles have a higher surface-to-volume ratio, making them more easily taken up by cells, and they possess different translocation potentials [79]. Most studies only report the average particle size or range rather than the complete distribution. A few studies that report the particle size distribution typically find that, in terms of quantity, the majority of particles are concentrated in the smaller size range (<100 µm), which is more significant from a toxicological perspective but also more challenging to precisely quantify [10]. Future studies must include detailed information on particle size distribution in order to conduct more advanced risk assessments.

## 4. Human Exposure and Health Implications

The health implications conferred below are primarily derived from in vitro studies, rodent models, and observational human studies examining correlations. While these studies provide biologically plausible mechanisms of action, they do not establish direct causality in populations exposed to environmental levels. These findings should be interpreted as indicating potential hazards rather than confirming risks at the population level.

### 4.1. Dietary Intake of MPs

MPs are introduced to the human body mainly through direct contamination pathways, one of the most critical exposure vectors being food and beverage packaging. MPs enter consumables through multiple routes; beverages, especially, are a significant vector for direct MP ingestion. Plastic tea bags can release over one billion particles per serving with brewing temperatures at 95 °C, while reusable bottles can shed millions of MPs through regular use [21,26]. The corresponding quantitative exposure assessment shows high daily intake burdens. An average adult intakes 39,000–52,000 MP particles per year from dietary sources alone; bottled water consumers could be exposed to up to 90,000 MPs/year—22.5 times higher than in tap water drinkers (~4000 MPs/year) [17,22]. The overall annual particle intake rises to 74,000–121,000 particles per person when including inhalation exposure. These estimates, which combine results from multiple studies using different methodologies, should be viewed as preliminary approximations with broad confidence intervals. They can be used for hazard ranking but are not sufficient for precise risk characterization.

These facts are likely to signify conservative assessments, as current analytical methods detect only a fraction of sub-micron particles, and vulnerable populations face disparate risks: infants using plastic feeding bottles ingest up to 4.55 million particles daily through formula preparation [21]. From a risk characterization perspective, infants and young children belong to a high-risk group due to a high intake-to-bodyweight ratio and developmental sensitivity, suggesting that existing exposure estimations may exceed the assumed protective margin for food contact materials. The ubiquity of packaging-derived contamination establishes processed foods and beverages as dominant exposure amplifiers in relation to environmental sources. Preparation of food also contributes as a major source of MPs; for example, plastic cutting boards can add ~49.5–50.7 g/person/year [38], as do poultry and fish (0.03 ± 0.04 and 2.6 ± 2.8 particles/g) [39]. Kitchen blenders also have the potential to release hundreds of millions of particles in one 30 s use, directly contaminating prepared food [41]. Daily activities, such as making tea, drinking bottled beverages, using plastic chopping boards, and using baby bottles, create low, diffuse exposures, combined with sporadic high doses. Furthermore, compared to other exposure pathways, dietary intake is the primary route of MP exposure in the human body, with the quantity of particles ingested being higher than those acquired through inhalation.

#### 4.1.1. Gastrointestinal Uptake and Cellular Damage

MP emissions from plastic tea bags and baby bottle sterilization are highly abundant; their small size enables them to migrate across biological barriers. MPs of varying sizes are released; their fate varies significantly. While larger particles (>130 µm) are likely excreted, smaller MPs and, particularly, NPs (≤130 µm) may cross the gastrointestinal epithelium, mainly via M-cells and Peyer’s patches [80]. This kind of transcellular passage initiates a series of pathological complexities; physical abrasion is responsible for injuring tight epithelial junctions, while internalized particles provoke oxidative stress through overproduction of reactive oxygen species (ROS) [81]. Consequently, mitochondrial dysfunction and lysosomal impairment disturb the cellular homeostasis, which triggers apoptosis [82,83]. Compared to larger-sized MPs, NPs showed enhanced bioactivity, directly compromising the membrane integrity and amplifying ROS generation. The resulting inflammation increases epithelial permeability, establishing a self-reinforcing cycle that accelerates MP translocation and systemic dissemination [22].

#### 4.1.2. Bioaccumulation and Systemic Health Impacts

Chronic exposure through packaged foods and bottled water stored for a long period comprises the continuous and low intake of MP particles. Even though the instant dose may be lower than in acute conditions, the persistence of this exposure brings a constant source for polymers and their key chemical additives. MPs act as a dual-threat vector through leaching from inherent additives such as bisphenol A, phthalates, and heavy metals (Cd and Pb) and the adsorption of environmental co-contaminants, including persistent organic pollutants (POPs). These free chemicals have endocrine-disrupting activities, with bisphenol A disrupting estrogen receptor signaling at concentrations as low as 1 nM, while phthalates impair testicular steroidogenesis [14]. The high surface-area-to-volume ratio of MPs boosts POP bioavailability compared to free aqueous forms and thereby expedites bioaccumulation in adipose tissue [84]. From a risk assessment perspective, chronic, low-dose exposure may be more significant from a toxicological perspective than short-term, high-dose exposure, mainly when pooled with long-term leaching and bioaccumulation effects.

Chronic exposure in mammalian models reveals multisystemic toxicity. Murine studies demonstrate metabolic dysregulation, categorized by hepatic steatosis and altered peroxisome proliferator-activated receptor gamma (PPARγ) expression, reproductive impairment evidenced by 30–45% reduced litter size and spermatogenesis arrest, and neurobehavioral deficits with impaired spatial memory. These changes are associated with microbiome disruption, as demonstrated by a >2.8× Firmicutes–Bacteroidetes ratio shift and mitochondrial dysfunction in blood–brain barrier endothelial cells [85]. Compared to plastic that has already aged in the environment and whose additives may have been consumed, MPs emitted from packaging materials by chemical leaching or mechanical fatigue have more primitive features and may, therefore, be more expected to release additives. This pathway illustrates the potential risk of chronic metabolic and endocrine disruption linked with consuming packaged food on a regular basis. It is important to note that the exposure concentrations and particle types (e.g., pristine polystyrene spherical particles) employed by many toxicology research institutions may not accurately represent the complex, low-dose, chronic exposure scenarios from dietary sources, which significantly limits the applicability of the findings for direct extrapolation to human health risks. The shape of the particles (fibers, fragments, spheres, and films) is another critical physicochemical characteristic that influences toxicity. The inflammatory potential and clearance rates of fibers from tea bags or synthetic textiles may differ from those of irregular fragments or spherical particles generated through abrasion [86,87]. However, most exposure assessments do not systematically quantify or report particle shape data, and toxicological studies often rely on spherical models, limiting our understanding of shape-specific effects in real-world environments. Therefore, incorporating shape analysis into routine characterization is needed.

#### 4.1.3. Microbiome Dysbiosis and Gut Barrier Impairment

MPs produced by mechanical friction, such as cutting boards and kitchen blenders, are usually large and irregular fragments and fibers. Their primary health effects are likely to be confined to the gastrointestinal tract rather than throughout the body. The irregular shape of these particles may contribute to physical abrasion on the intestinal epithelium, damage tight junctions, and induce a local inflammatory response [88]. Furthermore, these particles may cause an imbalance in the gut microbiota. Evidence from animal models suggests that the intake of MPs reduces beneficial symbiotic bacteria (such as *Ruminiclostridium* spp.) by 40–60% while promoting mucus-degrading opportunistic pathogens and endotoxin-producing deformation bacteria (Proteobacteria). This imbalance will disrupt microbial metabolism and the production of the short-chain fatty acids, which are critical in maintaining colonic cellular integrity, as well as the integrity of the gastrointestinal barrier [89,90]. Consequently, the abrasive particles resulting from the constant flow of food preparation may potentially disrupt gut homeostasis.

#### 4.1.4. Distinct Consideration of NPs

NPs are often released instantaneously with MPs from sources such as tea bags and blenders [26,41], posing unique challenges. Their smaller particle size facilitates greater biological uptake, with the potential to cross the intestinal barrier and even the blood–brain barrier. The toxicokinetic characteristics of NPs also differ from those of MPs [91]. However, due to analytical limitations in detecting particles smaller than 1 µm, the quantitative assessment of exposure to NPs remains highly uncertain. Similarly, the regulatory framework for NPs is still insufficient, with current focus primarily on metrics related to the mass or quantity of larger MPs. Future risk assessments will need to employ appropriate, size-sensitive methods to explicitly account for NPs as a distinct component.

### 4.2. Impact on Vulnerable Population

**Figure 5:**
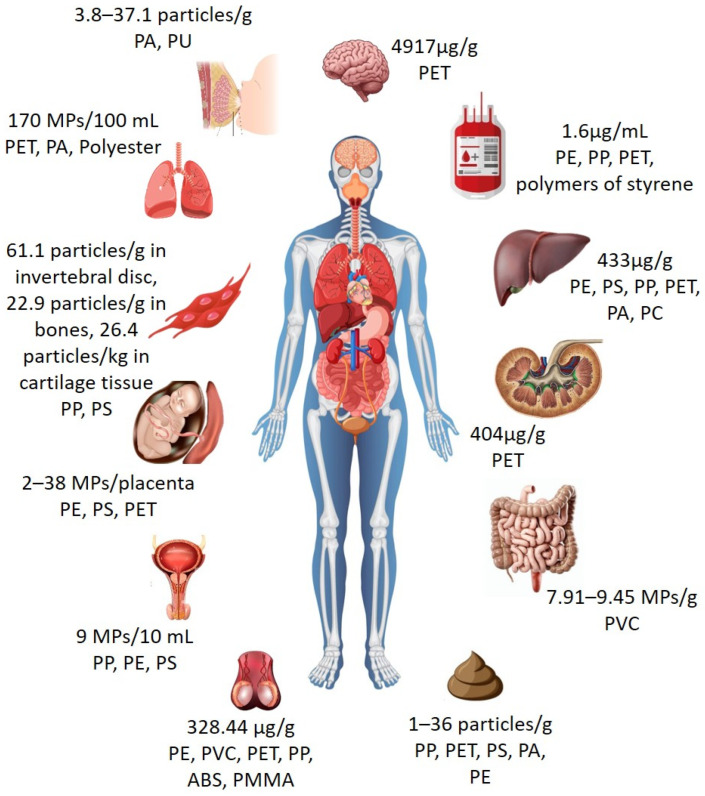
The figure describes the concentration of MPs reported in human organs with respect to polymer types.

Susceptible populations, particularly fetuses, infants, and pregnant women, are exposed to higher doses of MPs via development-stage-specific routes such as placental transfer, breastfeeding, and food contact materials [92]. Infants, children, and juveniles are exposed to MPs at the forefront of the MP point of discharge. Empirical data support extensive contamination of vital biological matrices, which include MP identification in 67% of human placental tissue samples [93]. Moreover, quantitative data on MPs in the placenta (12.2–33.5 particles/g), breast milk (3.8–37.1 particles/g), and infant feces (14.3–131.1 particles/g) have been established [94] (Table 2, Figure 5). Exposure also exists in neonates as MPs accumulate in meconia (the first newborn feces) and infant excrement [95]. The source identification of MPs, even in infants, is difficult due to the multisource influences of formula, dietary constituents, drinking water, and airborne particles [21,96,97,98].

The increased vulnerability of children is attributed to a high intake-to-mass ratio (3 to 4-fold higher than in adults); certain vulnerabilities in development, which include an immature detoxification system and blood-brain barrier; and possible dose magnification processes in mother–child interactions [99,100]. Evidence from animal studies shows that prenatal exposure to MPs triggers transgenerational effects, which include fetal metabolic predispositions (PPARγ dysregulation), neurodevelopmental changes with an over 40% decrease in brain-derived neurotrophic factor (BDNF) levels, and immune system predispositions such as T-helper2 (Th2) cytokine deviation. Corresponding challenges during pregnancy in human populations appear to be likely [101]. Prenatal–early life exposure level standards are an important concern in assessing lifetime exposure risks for such susceptible populations.

**Figure 6:**
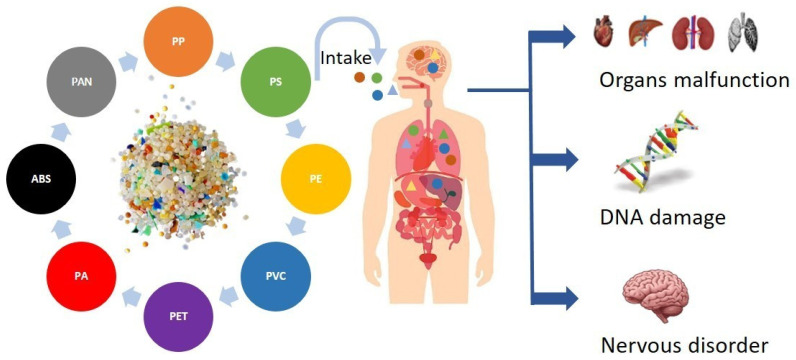
This figure shows that humans can be exposed to different MPs through ingestion, inhalation, and dermal contact, leading to neurological disorders, DNA damage, and organ dysfunction.

Elderly individuals also experience exacerbated MP-related health risks owing to natural age-related physiologic susceptibilities, such as immunity, chronic inflammation, and gut barrier dysfunction. While this is a partially acknowledged area of study in gerontology, inhalation testing indicates substantial pulmonary uptake, as shown by lung inflammation, which may be stimulated by MPs (Figure 6), as well as the detection of plastic fibers in cancerous lung tissue [102]. Pulmonary system assessments show widespread contamination, reflecting 170 particles/100 mL of PET, as well as polyamide (PA), in bronchoalveolar lavage fluid [103] (Table 2). However, neurological susceptibility is an even more pressing issue, as MPs <3 µm bypass the blood–brain barrier to decrease acetylcholinesterase function by >40% in addition to stimulating ROS, mediating oxidative damage to lipids, proteins, and DNA [91,104,105]. Simultaneously, contaminants in the urogenital system also reach 9 MPs per 10 mL (mostly PS, PP, and PE in fragment/fiber form <100 µm in size), suggesting systemic distribution [106] (Table 2; Figure 5). These multisystem exposures through environmental, dietary, and inhalation pathways build inflammatory cascades and cellular damage, possibly impacting elderly populations due to weakened detoxification capacity.

**Table 2:** MP occurrence in human organs and tissues, including particle shape, polymer types, size distribution, and study origin.

Human Organs	MP Shape	Polymer Composition	Size Distribution (μm)	MP Concentration	Identification and Quantification	Methodological Limitations	Confidence Level	Study Origin	References
Lungs	Fiber, fragment	Polyester, PET, PA	<100	170 MPs/100 mL	μ-Raman spectroscopy, SEM-EDS	Limited sample size, potential airborne contamination during sampling, fibers prone to secondary contamination	Moderate	Iran	[103]
Skeletal tissues	Fragments	PP, PS, ethylene venylacetate copolymer	159.5, 138.86, 87.5	61.1 ± 44.2 particles/g in intervertebral disc, 22.9 ± 15.7 particles/g in bones, 26.4 ± 17.6 particles/kg in cartilage tissue	μ-Raman spectroscopy, Stereo fluorescence microscopy	Complex tissue digestion, limited information on recovery efficiency	Moderate to high	China	[107]
Breast milk, placenta, infant feces	-	PA, PU	20–50	3.8 to 37.1 particles/g in breast milk, 12.2 to 33.5 particles/g in placenta, 14.3 to 131.1 particles/g in infant feces	LDIR	Cross-matrix comparison, potential maternal-to-infant transfer pathways inferred	Moderate	China	[94]
Lung tissue	-	PVC	20–100	14.19 ± 14.57 particles/g	LDIR, FTIR	Small cohort, post-mortem sampling may not represent general population	Moderate	China	[108]
Urinary system	Fiber, fragment	PP, PS, PE	<100	9 MPs/10 mL	μ-Raman spectroscopy	Low concentrations near detection limit, short residence time of MPs in urine uncertain	Moderate	Iran	[106]
Brain, kidney, liver	Pellets, rod-shaped	PET	1–5	4917 μg/g in brain, 404 μg/g in kidney, 433 μg/g in liver	ATR-FTIR, SEM-EDS	Extremely high values, limited sample number, potential contamination during digestion	Low to moderate	USA	[109]
Feces	-	PP, PS, PET, PE, PA, PC	20–800	1 to 36 particles/g	-	Absence of polymer-specific confirmation, reflects excretion rather than tissue accumulation	Low to moderate	USA	[95]
Intestine, tonsils	Fiber, fragment, film, spherical	PVC	20–100	7.91 ± 7.00 to 9.45 ± 13.13 MPs/g in intestine, 6.03 ± 7.37 MPs/g in tonsils	LDIR, FTIR	Limited anatomical coverage, potential cross-contamination during dissection	Moderate	China	[108]
Placenta	Fiber, fragment	PS, PE, PET	<10	2 to 38 MPs/placenta	μ-Raman spectroscopy	Low particle counts; sampling and digestion steps critical	Moderate to high	Iran	[110]
Testes	-	PE, PVC, PET, PP, ABS, PMMA	-	328.44 μg/g	Py-GC/MS	Mass-based measurement, particle number not quantified	Moderate	USA	[111]
Blood	-	PET, PE, polymers of styrene, PP	-	1.6 μg/mL	ATR-FTIR, μ-FTIR, Raman spectroscopy	Low concentrations, short circulation time, strict contamination control required	High	Netherlands	[112]

Note: The sign “-” indicates unspecified. Reported concentrations in human tissues and biological samples vary widely due to differences in analytical techniques, sample digestion protocols, and contamination control. These findings reflect detection rather than demonstrated toxicological thresholds and should not be interpreted as direct indicators of health risk.

## 5. Industry Initiatives and Mitigation Strategies

This review specifies the evidence of the direct release of MPs from food contact materials that forms a significant pathway of human exposure. Because of this challenge, addressing the issue would need a multifaceted approach, from simply unfolding current initiatives to proposing a positive, multilayered response framework. In this section, an understanding of the mechanistic interpretation of MP release is proposed with actionable solutions, covering aspects such as simple material design, regulatory invention, and consumer behavior in a three-tiered defense system.

### 5.1. Material Science and Safe by Design

Fundamental and effective mitigation means stopping the emission of MPs through a “safe-by-design” perspective during the manufacturing and design phase. The technique directly addresses release mechanisms such as thermal stress, mechanical wear, and chemical leaching. The manufacturing of polymer materials can meaningfully improve the tolerance of a material [113]; for example, the improvement of polymers with a higher degree of crystallinity and fracture toughness can reduce fragmentation due to mechanical wear, such as cutting boards and blenders [41]. Similarly, plastics with good thermal stability against short circuit and crosslinking structures should be prepared to minimize degradation and particle shedding from baby bottles and teabags at high temperatures. In addition, surface engineering practices, such as the use of durable, inert ceramic or diamond-like carbon coatings, can form a protective barrier over the external surface area of the plastic, thus reducing direct wear and loss [114]. Another key parallel direction of development is the optimization of additive systems toward non-removable, chemically bonded alternatives to traditional plasticizers and stabilizers, considerably decreasing toxicological risks even in the case of particle release [62].

### 5.2. Standards, Certification, and Informed Choices

A keystone of reliable research on MPs depends on contamination control throughout the sampling, processing, and analysis processes, as synthetic fibers are ubiquitous in both air and laboratory environments. Many early studies failed to adequately report procedural gaps, potentially leading to overestimation of reported concentrations. Furthermore, inter-laboratory comparison studies have shown that even with standardized samples, there is significant variability between different laboratories, highlighting the need for established, validated operating procedures and reference materials [98]. Future regulatory standards must mandate stringent pollution control measures and the systematic reporting of quality assurance/quality control (QA/QC) data. While ideal “safe-by-design” materials have not yet been fully adopted, the launch of a sound regulatory framework will help guide the industry and consumers. Presently, strategies at the global level remain fragmented, with no consistent, harmonized metrics to measure the release of MPs [115,116]. We proposed a “dual-track” approach to regulatory development. Firstly, there is an urgent need for international regulatory leading bodies, such as the U.S. Food and Drug Administration (FDA), the European Food Safety Authority (EFSA), and the State Administration for Market Regulation (SAMR) in China, to develop and implement harmonized testing protocols capable of simulating realistic conditions of use. The testing protocols need to be product-specific, such as a tea bag test that quantifies MNP release at brewing temperatures, a cutting board wear test that simulates food preparation processes, and a bottle sterilization test that replicates the preparation process for infant formula. Secondly, based on these harmonized data, we introduced the novel concept of the “microplastic release potential” (MRP) label, which should be designed for consumers and works like energy efficiency ratings, allowing for the categorization of products based on their MP release levels, such as categories A to D. This system will offer informed choices for consumers and create market-driven incentives for manufacturers to develop low-emission-potential products.

### 5.3. Behavioral Interventions and Smart Use

For current quantities of plastic products, instantaneous risk prevention might be accomplished by employing behavior modifications grounded in scientific support. The recommendations could be inferred from the processes of MP release discussed in this review. To reduce the release of MPs due to thermal stress, an individual could prepare infant formula by first cooling boiled water to below 70 °C in a non-plastic-material pot, prior to packaging it in a plastic material bottle. This can also be accomplished during the brewing of plastic-based tea bags by ensuring that the water temperature is cooler and thus safer slightly prior to pouring it to brew the tea. Furthermore, by avoiding repetitive processes during blender usage when dealing with more solid food constituents, an individual might reduce MP exposure by employing a wooden or bamboo cutting board in place of a plastic-based cutting board [38]. Moreover, making sensible choices such as choosing paper, cotton, or loose-leaf tea alternatives to plastic tea bags and using glass or stainless-steel containers for food storage and heating can directly avoid the primary sources of MPs [43]. Public health campaigns that emphasize these basic and actionable measures can serve as an instant and effective way to reduce individual exposure before more systemic changes can be fully implemented.

## 6. Conclusions and Future Perspectives

While plastics have provided significant societal benefits, their pervasive contamination now permeates the global food chain. This review assembles strong evidence of the causative route of human exposure as the direct emission of MPs into the human food chain from food contact materials and kitchen utensils, and it demonstrates how common human activities, such as brewing tea, preparing baby formula, chopping vegetables, or stirring drinks, constitute central emission sources of MPs. The emission of MPs from certain processes, such as thermal stress in the course of heating practices, wear and tear in the course of usage, and leaching processes for stored products, implies chronic exposure to high concentrations of MPs, which have only lately been studied regarding their toxicological impacts on human health. Toxicological information based on animal models suggests that MPs have potential influences in eliciting oxidative stress, gastrointestinal barrier dysfunction, and inflammation, constituting a significant threat to susceptible populations such as pregnant women, as well as infants.

This study highlights that these risks are not equal but instead depend upon material fragility and stress intensity. A long-term and efficient strategy is to reform materials science, moving toward a new safe-by-design model and taking up a novel, more resistant generation of polymers. This scientific improvement needs to be conducted in the regulatory domain by creating standardized testing procedures and bringing a novel consumer end in the form of a standard MRP. Moreover, immediate risk reduction can be accomplished through public empowerment by promoting the avoidance of plastics at high temperatures and promising the choice of safer alternative materials. Future research must prioritize the development and standardization of analytical methods capable of distinguishing and quantifying MPs and NPs within complex matrices. The risk assessment framework should also be continuously refined to fully account for the differences in the properties and toxicological effects of these particles of different sizes. While this review synthesizes relevant evidence showing that direct release from food contact materials may be an important pathway for human exposure to MPs, it is important to note that global-scale exposure estimates often rely on extrapolations from limited product studies and specific geographic regions.

Looking forward, the future course of action should be anchored through focused research. The current burning issues are the optimization of analytical methods concerning the most difficult MPs to detect in complex food systems; encouraging the advancement of toxicology research toward the adoption of more realistic low-dose models of exposure; and the cause-and-effect relationship concerning the epidemic diseases of humans and health effects. The issue originates from the current stage of scientific discovery in the world of policy intervention, which is the domain of governments and international organizations, and it requires greater regulation. The United Nations Environment Assembly resolution on the adoption of a globally binding instrument concerning plastic pollution, the European Union’s adoption of the Single-Use Plastics Directive, and the REACH restrictions regarding the intentional addition of MPs indicate significant instances of this issue being considered. Consequently, regarding further increasing the efficiency of future scientific research, there is a need to promote regulatory procedures through the provision of scientific information to further assess the usefulness of intervention policies and initiate a very sound foundation for the adoption of a circular and, thus, safe-by-design plastics economy.

## Data Availability

No new data were created or analyzed in this study. Data sharing is not applicable to this article.

## Abbreviations

The following abbreviations are used in this manuscript:

MPs	Microplastics
NPs	Nanoplastics
PP	Polypropylene
PS	Polystyrene
PET	Poly-ethylene terephthalate
PU	Polyurethane
PE	Polyethylene
PVC	Polyvinyl chloride
PA	Polyamide
PAA	Polyacrylic acid
PEVA	Polyethylene vinyl acetate
PAM	Polyacrylamide
PEA	Poly (ester-amide)
ABS	Acrylonitrile butadiene styrene
LDPE	Low-density polyethylene
PMMA	Polymethyl methacrylate
PPSU	Polyphenylene sulfone
PVA	Polyvinyl acetate
SAN	Styrene–acrylonitrile
PC	Polycarbonate
EDI	Estimated dietary intake
FTIR	Fourier-transform infrared spectroscopy
ATR	Attenuated total reflectance
SEM	Scanning electron microscopy
EDX	Energy-dispersive X-ray spectroscopy
EDS	Energy-dispersive spectroscopy
XPS	X-ray photoelectron spectroscopy
LDIR	Laser direct infrared spectroscopy
Py-GC/MS	Pyrolysis gas chromatography–mass spectrometry
BDNF	Brain-derived neurotrophic factor
Cd	Cadmium
Pb	Lead
Ca^2+^	Calcium ion
HCO_3_^−^	Bicarbonate ion
Fe^3+^	Ferric ion
Cu^2+^	Cupric ion
Fe_2_O_3_	Ferric oxide
POPs	Persistent organic pollutants
AOPs	Advanced oxidation processes
Th2	T-helper2
ROS	Reactive oxygen species
PPARγ	Peroxisome proliferator-activated receptor gamma
MRP	Microplastic release potential
EFSA	European Food Safety Authority
FDA	Food and Drug Administration
SAMR	State Administration for Market Regulation
